# Role of calcium desensitization in the treatment of myocardial dysfunction after deep hypothermic circulatory arrest

**DOI:** 10.1186/cc13071

**Published:** 2013-10-20

**Authors:** Alessio Rungatscher, Seth Hallström, Alice Giacomazzi, Daniele Linardi, Elisabetta Milani, Maddalena Tessari, Giovanni Battista Luciani, Tiziano M Scarabelli, Alessandro Mazzucco, Giuseppe Faggian

**Affiliations:** 1 Department of Surgery, Division of Cardiac Surgery, University of Verona, Pz.le Stefani 1, 37126 Verona, Italy; 2 Institute of Physiological Chemistry, Medical University of Graz, Graz, Austria; 3 Center for Heart and Vessel Preclinical Studies, St. John Hospital and Medical Center, Wayne State University Medical School, Detroit, MI, USA

## Abstract

**Introduction:**

Rewarming from deep hypothermic circulatory arrest (DHCA) produces calcium desensitization by troponin I (cTnI) phosphorylation which results in myocardial dysfunction. This study investigated the acute overall hemodynamic and metabolic effects of epinephrine and levosimendan, a calcium sensitizer, on myocardial function after rewarming from DHCA.

**Methods:**

Forty male Wistar rats (400 to 500 g) underwent cardiopulmonary bypass (CPB) through central cannulation and were cooled to a core temperature of 13°C to 15°C within 30 minutes. After DHCA (20 minutes) and CPB-assisted rewarming (60 minutes) rats were randomly assigned to 60 minute intravenous infusion with levosimendan (0.2 μg/kg/min; n = 15), epinephrine (0.1 μg/kg/min; n = 15) or saline (control; n = 10). Systolic and diastolic functions were evaluated at different preloads with a conductance catheter.

**Results:**

The slope of left ventricular end-systolic pressure volume relationship (Ees) and preload recruitable stroke work (PRSW) recovered significantly better with levosimendan compared to epinephrine (Ees: 85 ± 9% vs 51 ± 11%, *P*<0.003 and PRSW: 78 ± 5% vs 48 ± 8%, *P*<0.005; baseline: 100%). Levosimendan but not epinephrine reduced left ventricular stiffness shown by the end-diastolic pressure-volume relationship and improved ventricular relaxation (Tau). Levosimendan preserved ATP myocardial content as well as energy charge and reduced plasma lactate concentrations. In normothermia experiments epinephrine in contrast to Levosimendan increased cTnI phosphorylation 3.5-fold. After rewarming from DHCA, cTnI phosphorylation increased 4.5-fold in the saline and epinephrine group compared to normothermia but remained unchanged with levosimendan.

**Conclusions:**

Levosimendan due to prevention of calcium desensitization by cTnI phosphorylation is more effective than epinephrine for treatment of myocardial dysfunction after rewarming from DHCA.

## Introduction

Deep hypothermic circulatory arrest (DHCA) is widely used during repair of the aortic arch and congenital heart defects as a method of cerebral protection. However, the effect of myocardial ischemia-reperfusion injury combined with hypothermia-rewarming has important clinical implications and remains a significant contributor to perioperative morbidity and mortality [1].

Hypotension and low cardiac output are common after rewarming and up to 50 to 80% of patients have hemodynamic instability [2].

Because of the limited knowledge of the pathophysiology of hypothermia- and rewarming-induced cardiac dysfunction either in the setting of cardiac surgical intervention or in accidental hypothermia, guidelines for pharmacological treatment are missing [3]. Among the mechanisms underlying cardiac dysfunction after hypothermia-rewarming, a pivotal role has been assigned to Ca^2+^ overload and the paradoxical myofilaments Ca^2+^ desensitization. Ca^2+^-dependent interactions among myofilament regulatory proteins primarily control cardiac muscle contraction. Among them, troponin and tropomyosin confer not only Ca^2+^ sensitivity to contraction, but also modulation by phosphorylation of troponin I (cTnI) [4]. The latter represents a well-established mechanism for decreased myofilament Ca^2+^ sensitivity and could be mediated by phosphokinase A activated by β-adrenergic stimulation [5].

Thus, agents that target the regulatory apparatus by increasing the calcium sensitivity without deleterious effects on the intracellular calcium concentration or oxygen consumption are useful in the treatment of cardiac dysfunction. Epinephrine is currently used in the treatment of hypothermia-induced cardiac dysfunction [6]. However, the efficacy of epinephrine in this setting has been questioned [7-9].

Agents that exert a positive-inotropic effect, by directly acting on thin filament regulatory proteins or on cross-bridge cycling, are commonly referred to as calcium sensitizers. Among them, levosimendan, in contrast to epinephrine, improves ventricular function without increasing oxygen demand [10]. We previously demonstrated that levosimendan compared to epinephrine has better inotropic and lusitropic effects when administrated during rewarming from DHCA [11].

The present study was designed to investigate treatment with levosimendan or epinephrine after rewarming from DHCA when acute myocardial dysfunction represents a clinical problem. We aimed to investigate the effects of levosimendan and epinephrine on calcium desensitization mediated by cTnI phosphorylation. Moreover, we purposed to evaluate the acute overall hemodynamic, biochemical and metabolic effects of the two pharmacologic agents after rewarming from DHCA.

## Methods

The Institutional Animal Care and Use Committee of the University Animal Research Laboratory approved this study. All animals received standardized care in accordance with the National Institute of Health Guidelines. Adult male Wistar rats (400 to 450 g, Harlan, Udine, Italy) were used for all experiments.

### Animal preparation

Rats were anesthetized with 5% isoflurane in 50% O_2_ in a plastic induction box. After orotracheal intubation with a 14G cannula, the animals were mechanically ventilated (Harvard Model 687, Harvard Apparatus, Holliston, MA, USA). The tidal volume was 7 ml/kg and the respiratory rate was 50 to 60 breaths/minute with an air-oxygen mixture (FiO2 = 0.5). Ventilation was adjusted to keep an arterial carbon dioxide tension (PaCO_2_) of 35 to 45 mm Hg. Anesthesia was maintained with isoflurane 2% and pancuronium-bromide (2 mg/kg iv) was administered for complete muscle relaxation. Adequate anesthesia was monitored by the withdrawal response to a paw pinch and respiration monitoring. Rats were secured supine on a heating board. ECG was monitored using limb leads. A thermocouple microprobe was inserted into the left femoral artery and advanced into the descending aorta for the measurement of blood temperature. The left femoral artery was cannulated with a heparinized 24-G Teflon catheter to monitor systemic arterial pressure and to collect arterial blood for lactate and gas analysis.

### Cardiopulmonary Bypass (CPB) model

Central cannulation was performed as previously described [12]. In brief, after complete sternotomy, a venous cannula (a modified four-hole 16 gauge Angiocath catheter, Delta Med, New York City, NY, USA) was advanced into the right atrium using a right trans-superior vena cava approach, allowing excellent drainage. The left common carotid artery was cannulated using an 18-gauge catheter advanced to the aortic arch and connected to the arterial perfusion line for the CPB circuit. Full heparinization (500 IU/kg) was assured after surgical preparation and immediately before CPB initiation.

CPB was set up as previously described [12]. The setup consisted of a venous reservoir, a roller pump, a hollow-fiber oxygenator (Sorin, Mirandola, Italy), and a vacuum regulator with an applied pressure of 30 mmH_2_O to facilitate venous drainage, all connected by 1.6 mm internal diameter plastic tubing. Total priming volume was 9.5 mL, the gas exchange surface was 450 cm^2^, and the heat exchange surface was 15.8 cm^2^.

### Study design

CPB was instituted at a flow rate of 120 mL/kg/min. A core temperature of 15°C to 13°C was achieved over 30 minutes using CPB-assisted cooling. The roller pump was turned off and DHCA, as confirmed by asystole and lack of measurable mean arterial pressure, was maintained for 20 minutes at 13°C to 15°C.

With the reinstitution of CPB, rewarming started at a flow rate of 100 mL/kg/min. CPB inflow rate was gradually increased, reaching the full rate of 120 mL/kg/min at the end of rewarming at 36°C in a period of 60 minutes. The temperature gradient between the CPB circuit and body core did not exceed 10°C. After full rewarming, the remaining priming volume was reinfused and animals were weaned from CPB (T1). Then animals were randomly assigned to treatment with levosimendan (0.2 μg/kg/min) or epinephrine (0.1 μg/kg/min) or saline as control. Infusion started immediately after hemodynamic recording and blood sampling post-weaning from CPB (T1) and lasted for 60 minutes in normothermia at 36°C to 37°C (Endpoint = T2).

### Hemodynamic analysis

Hemodynamic parameters were collected continuously during the experiments with a 2 F micro-tip pressure-volume conductance catheter (SPR-838; Millar Instruments, Inc., Houston, TX, USA) inserted into the right carotid artery and advanced into the left ventricle. Signals were continuously recorded at a sampling rate of 1,000 samples/s using a P-V conductance system (MPVS-400; Millar Instruments, Inc.), stored, and displayed on a personal computer by the PowerLab Chart5 Software System (AD Instruments, Colorado Springs, CO, USA). With the use of a special pressure-volume analysis program (PVAN; Millar Instruments, Inc.), heart rate (HR), mean arterial pressure (MAP), maximal slope of the systolic pressure increment (+dP/dt) and the diastolic pressure decrement (−dP/dt), and time constant of left ventricular pressure decay (Tau, according to the Weiss method) were computed and calculated. Stroke volume (SV) and cardiac output (CO) were calculated and corrected according to *in vitro* and *in vivo* volume calibrations using PVAN software [13].

In addition to the above parameters, left ventricle pressure-volume relations were measured by transiently occluding the inferior vena cava (reducing preload) under the diaphragm by tying a snare suture around the vein at baseline condition (T0), after CPB weaning (T1) and at the end of 60 minutes of infusion treatment (T2) [13].

### Biochemical analysis

After euthanasia with a potassium bolus freeze-clamped left ventricle myocardial biopsies were snap-frozen. In particular, the tip of the freeze-clamp tong was pre-cooled in liquid nitrogen prior to taking the biopsies and thereafter the samples were stored at −80°C (protein isolation from tissue extracts) or in liquid nitrogen (high energy phosphates) until analysis.

For Western blot analysis of cTnI phosphorylation, cardiac specimens were pulverized in liquid nitrogen and homogenized in ice-cold lyses buffer (20 mM Hepes, 420 mM NaCl, 1 mM EDTA, 1 mM EGTA, 1% NP-40, 20% glycerol) containing a protease inhibitor cocktail (Roche Diagnostics, Monza, Italy) and a phosphatase inhibitor cocktail (Sigma-Aldrich, St. Louis, MO, USA).

The homogenates were centrifuged (12,000 rpm) for 30 minutes at 4°C, supernatants were recovered, snap-frozen in liquid nitrogen and stored at −80°C. Total protein content was determined with the Bradford method. Equal amounts of each sample were fractionated on 10% polyacrylamide gels by SDS-PAGE and transferred to polyvinylidene difluoride membranes (Millipore, Bedford, MA, USA). Membranes were blocked with 5% BSA (Albumin from Bovine Serum, Sigma-Aldrich) in TBS-0.1% Tween20, and incubated overnight with specific primary antibodies for either phospho-cTnI (Ser23/24) (1:1,000, Cell Signaling Technology, Danvers, MA, USA) or total cTnI (1:1,000, Cell Signaling Technology) at 4°C. A horseradish peroxidase conjugated secondary antibody (Amersham Biosciences, GE Healthcare Europe, Munich, Germany) was used to detect the blots by standard chemiluminescence substrate (LiteAblot PLUS reagent, Euroclone, Siziano, Italy) on Kodak films (Carestream Health Inc., Rochester, NY, USA). Bands were quantified by ImageJ software (National Institutes of Health, Bethesda, MD, USA).

For energy status determination, the sample preparation and high-performance liquid chromatography (HPLC) measurement of ATP, ADP, AMP and phosphocreatine, as well as hypoxanthine and xanthine, were performed as previously described [12]. A piece of frozen tissue (50 to 100 mg) was homogenized with a micro-dismembranator and deproteinized with 400 μL of 0.4 mol/L perchloric acid. After centrifugation (12,000 g), 150 μL of the acid extract was neutralized with 15 to 20 μL of 2 mol/L potassium carbonate (4°C). The supernatant (20 μL injection volume), obtained after centrifugation, was used for HPLC analysis. The pellets of the acid extract are dissolved in 1 mL of 0.1 mol/L sodium hydroxide and further diluted 1:10 with physiological saline for protein determination (BCA Protein Assay, Pierce; Pierce Biotechnology, Rockford, IL, USA). High energy phosphates (ATP, ADP, AMP and phosphocreatine), hypoxanthine and xanthine were measured by HPLC as previously described [14]. In brief, separation was performed on a Hypersil ODS column (5 μm, 250 × 4 mm I.D.) using an L-2200 autosampler, two L-2130 HTA pumps and an L-2450 diode array detector (all: VWR Hitachi, VWR, Vienna, Austria). Detector signals (absorbance at 214 and 254 nm) were recorded, and program EZchrom Elite (VWR) was used for data requisition and analysis.

Energy charge was calculated using the following formula: (ATP + 0.5ADP)/(AMP + ADP + ATP).

Arterial blood samples were collected to measure lactate concentration.

### Statistical analysis

The means ± SD are given. For between-group comparisons, analysis of variance with the Bonferroni *post hoc* test was used. An unpaired two-tailed Student *t-*test or paired *t* test was employed to evaluate differences between groups and within groups versus the baseline, respectively. The statistical software GraphPad Prism 5.0 (GraphPad Software Inc., San Diego, CA, USA) was used. A *P*-value of <0.05 was considered to be significant.

## Results

Fifty rats were used for this study; 40 completed the experimental protocol and were included. Ten animals were excluded, including five rats due to their inability to wean from CPB and five due to instrumentation or technical failure during animal preparation.

Among the three studied groups no differences in baseline hemodynamic parameters were observed (Table 1). A marked reduction compared to baseline normothermia was seen in most hemodynamic parameters after rewarming from DHCA (Table 1).

**Table 1:** Hemodynamic parameters at different time points

		**Control**	**Epinephrine**	**Levosimendan**
**HR** bpm	T0	360 ± 16	354 ± 13	342 ± 12
	T1	340 ± 9	346 ± 7	322 ± 9
	T2	352 ± 11	362 ± 11	355 ± 10
**MAP** mmHg	T0	130 ± 11	127 ± 12	129 ± 13
	T1	92 ± 9	85 ± 8	87 ± 8
	T2	85 ± 6*	164 ± 13*	112 ± 10
**LVESP** mmHg	T0	95.2 ± 4.3	92.2 ± 6.4	93.4 ± 5.7
	T1	66.2 ± 7.4	58.4 ± 5.1	63.5 ± 6.0
	T2	72.4 ± 8.9*	104.3 ± 9.2	98.5 ± 4.7
**LVEDP** mmHg	T0	8.2 ± 0.8	8.0 ± 0.5	8.6 ± 0.6
	T1	12.2 ± 1.4	11.8 ± 2.1	12.5 ± 1.8
	T2	13.4 ± 0.9*	11.6 ± 0.8*	8.1 ± 0.5
**SV** μL	T0	65 ± 10	63 ± 9	63 ± 6
	T1	23 ± 8	25 ± 9	23 ± 9
	T2	25 ± 10*	41 ± 9*	58 ± 7
**CO** ml/min	T0	43 ± 3	47 ± 5	45 ± 5
	T1	15 ± 6	13 ± 8	14 ± 5
	T2	16 ± 10*	21 ± 5*	38 ± 7
**CI** ml/min/100 g BW	T0	9.02 ± 1.92	9.02 ± 1.92	10.8 ± 1.22
	T1	4.01 ± 0.51	4.21 ± 0.48	4.21 ± 0.48
	T2	4.35 ± 0.60*	4.95 ± 0.73*	9.95 ± 1.43
**dP/dt**_**max**_ mmHg/s	T0	7,305 ± 184	7,427 ± 193	7,289 ± 201
	T1	4,667 ± 145	4,588 ± 210	4,644 ± 185
	T2	4,850 ± 174*	7,171 ± 189	7,221 ± 174
**dP/dt**_**min**_ mmHg/s	T0	7,672 ± 306	7,511 ± 285	7,622 ± 297
	T1	4,856 ± 288	4,992 ± 199	4,741 ± 283
	T2	4,510 ± 190*	5,701 ± 189*	7,485 ± 190
**TPRI** mmHg/ml/min/100 g BW	T0	2.78 ± 0.22	2.63 ± 0.18	2.57 ± 0.25
	T1	1.98 ± 0.19	1.88 ± 0.21	1.90 ± 0.23
	T2	2.12 ± 0.25*	5.70 ± 0.70*	2.60 ± 0.31

(T0 = baseline; T1 = end of CPB-assisted rewarming; T2 = end of 60 min intravenous infusion).

CI, cardiac index; CO, cardiac output; dP/dtmax and dP/dtmin maximal slope of the systolic pressure increment and the diastolic pressure decrement, respectively; HR, heart rate; LVESP, LV end-systolic pressure; LVEDP, LV end-diastolic pressure; MAP, mean arterial pressure; SV, stroke volume; TPRI, total peripheral resistance index.

Data are expressed as mean ± SD *P<0.05 T2 vs T0.

**Figure 1:**
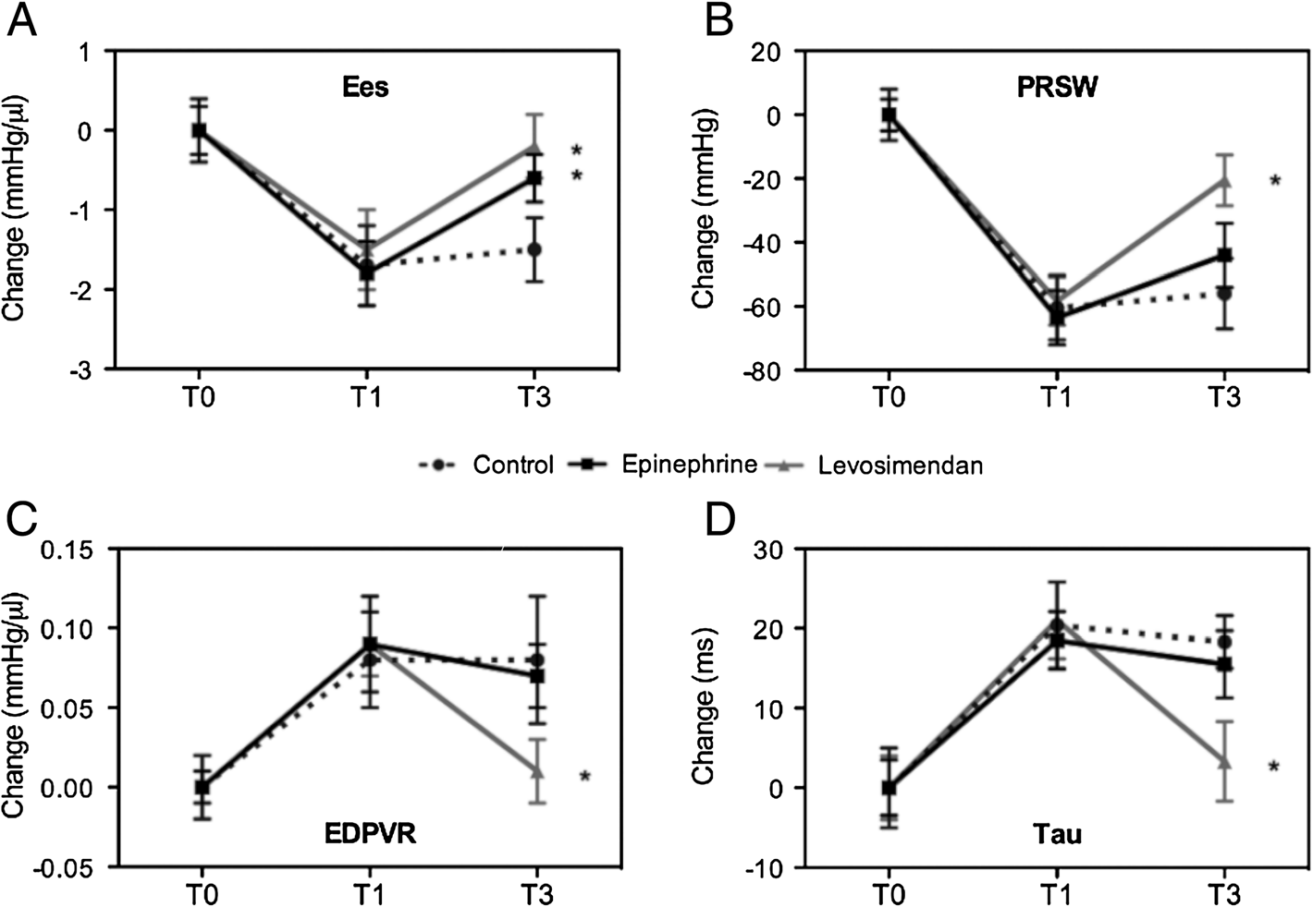
**Preload-independent hemodynamic parameters.** Systolic (Ees, PRSW; panel **A**, **B**) and diastolic (EDPVR, Tau; panel **C**, **D**) functions at baseline condition (T0), after CPB-assisted rewarming (T1) and after 60 minutes infusion of saline (control), levosimendan or epinephrine (T2). Values represent absolute changes (mean ± SD). EDPVR, end-diastolic pressure-volume relationship; Ees, slope of end-systolic pressure-volume relationship; PRSW, preload recruitable stroke work; Tau, time constant of left-ventricular pressure decay (Weiss method). *P<0.05 vs control.

Functional indices derived from pressure-volume analysis at different preloads are less influenced by loading conditions. In particular, Ees and PRSW were calculated as load-independent indexes of left ventricular contractility. Ees was significantly higher after both levosimendan and epinephrine infusion than control, indicating a steeper end-systolic pressure-volume relationship and a better systolic performance (Figure 1). Only levosimendan significantly improved PRSW; the slope was steeper after levosimendan compared to epinephrine treatment, indicating an improved systolic function determined by levosimendan in this setting (Figure 1). Compared with the corresponding control animals, the overall PRSW values did not reach statistical significance in the epinephrine group (Figure 1).

The diastolic function after rewarming was described by the left ventricular relaxation index (Tau) and the stiffness index end-diastolic pressure-volume relationship (EDPVR). Only levosimendan significantly improved EDPVR and Tau (Figure 1).

**Figure 2:**
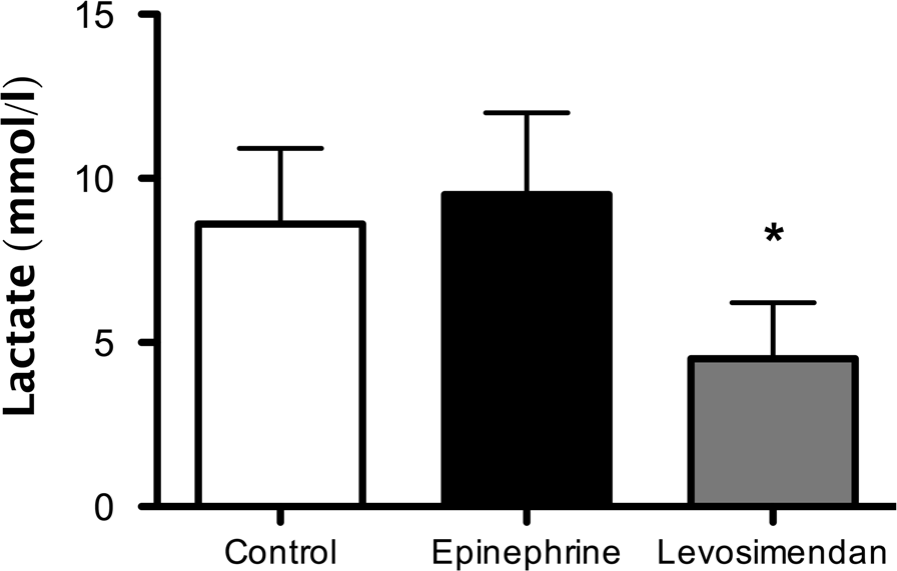
**Lactate plasma levels.** Levosimendan, but not epinephrine, reduced lactate production at T2 (the end of the 60-minute intravenous infusion). Data are expressed as mean ± SD. Symbols indicate intergroup statistical differences (*P<0.01 vs epinephrine and control).

Lactate levels were significantly higher after treatment with epinephrine as well as in the control group than after levosimendan infusion (8.2 ± 3.8 mmol/L and 9.3 ± 4.4 vs 4.1 ± 3.2 mmol/L respectively; *P*<0.001) (Figure 2).

**Figure 3:**
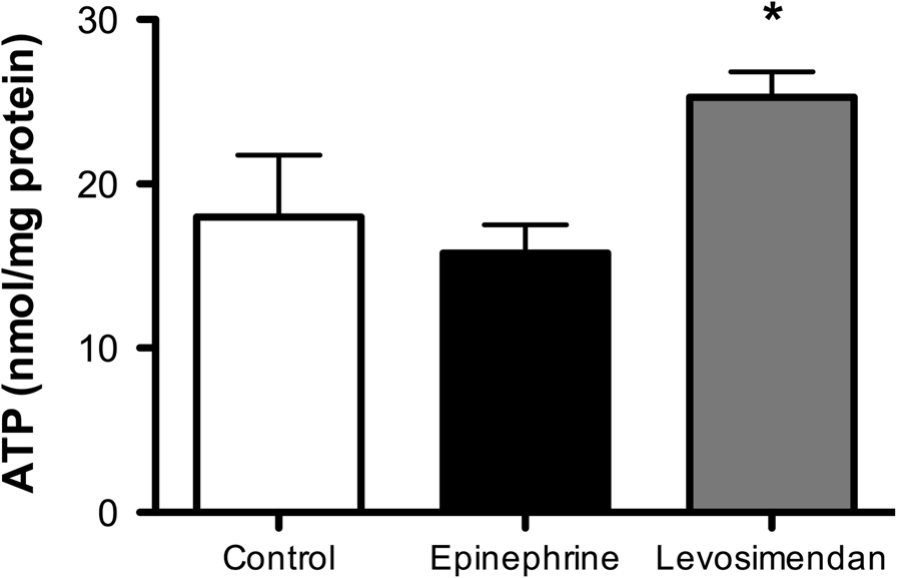
**High energy phosphates status: ATP myocardial content (nmol/mg).** Levosimendan improved myocardial ATP content at T2 (end of the 60-minute intravenous infusion). Data are expressed as mean ± SD. The symbol indicates intergroup statistical differences (*P<0.05 vs epinephrine and control).

**Figure 4:**
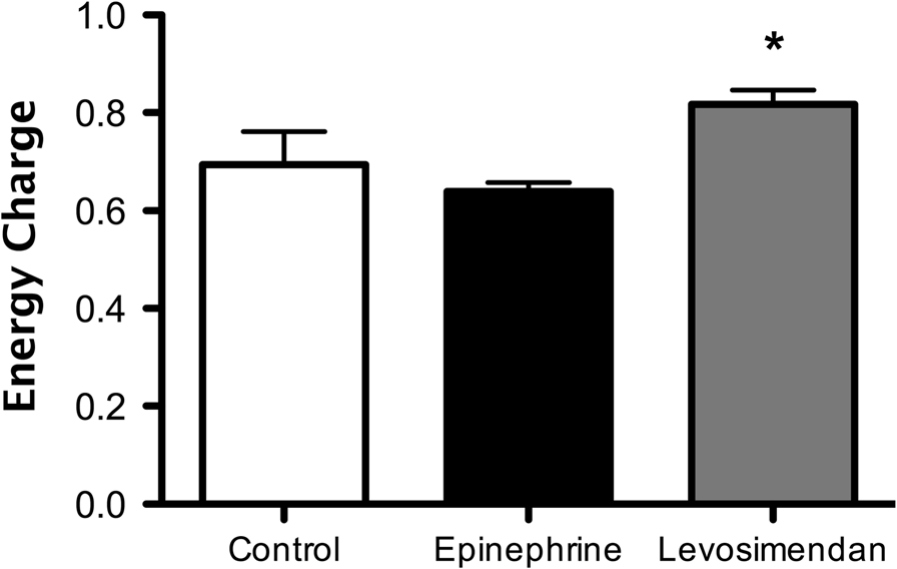
**High energy phosphates status: energy charge (ATP + 0.5 ADP)/(AMP + ADP + ATP).** Levosimendan ameliorated myocardial energy charge at T2 (end of the 60-minute intravenous infusion). Data are expressed as mean ± SD. The symbol indicates intergroup statistical differences (*P<0.01 vs epinephrine).

In addition, levosimendan better preserved the high-energy phosphate status of the left ventricle compared to epinephrine. The levosimendan group showed a significantly higher ATP content (25.25 ± 1.91 vs 15.8 ± 2.11 nmol ATP/mg protein; *P*<0.001) (Figure 3) and energy charge (0.80 ± 0.03 vs 0.65 ± 0.03; *P*<0.001; Figure 4). The high energy phosphate, hypoxanthine and xanthine values are summarized in Table 2. In line with the above findings, the epinephrine group had significantly higher AMP values compared to the levosimendan- and control group.

**Table 2:** High-energy phosphates, hypoxanthine and xanthine myocardial content at T2 (end of the 60-minute intravenous infusion)

**(nmol/mg protein)**	**Control**	**Epinephrine**	**Levosimendan**
**Phosphocreatine**	14.30 ± 7.92	6.78 ± 6.04	11.57 ± 0.76
**ATP**	18.65 ± 5.63	15.80 ± 2.11	25.25 ± 1.91*
**ADP**	8.74 ± 1.47	10.92 ± 2.12	9.63 ± 0.85
**AMP**	3.26 ± 1.92	6.26 ± 1.52^**§**^	2.64 ± 0.42°
**Hypoxanthine**	0.42 ± 0.07	0.33 ± 0.15	0.48 ± 0.03
**Xanthine**	0.43 ± 0.09	0.56 ± 0.19	0.38 ± 0.00

Data are expressed as mean ± SD. *P<0.001; °*P*<0.01 vs epinephrine. ^§^*P*<0.05 vs control.

**Figure 5:**
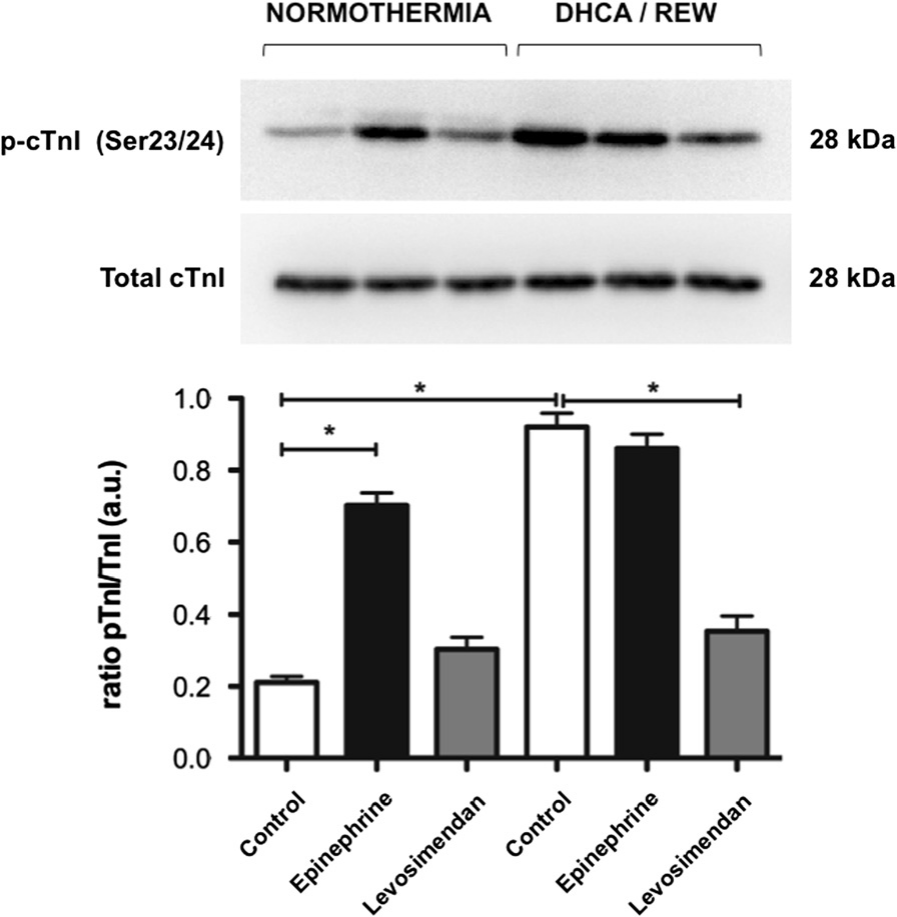
**Phosphorylation of cardiac Troponin I (cTnI).** Western blot analysis were performed on myocardial biopsies of normothermic controls (separate series) and after rewarming from DHCA. Epinephrine determined a significant phosphorylation of cTnI in both conditions. Rewarming from DHCA produced a similar phosphorylation. Levosimendan did not cause phosphorylation of cTnI during normothermia and prevented the phosphorylation after rewarming from DHCA. Values determined by densitometry of phosphorylated-cTnI (p-cTnI) over the relative expression of total cTnI. Data are shown here as mean ± SEM (n = 5 per group). Symbol indicates intergroup statistical differences (*P<0.05). DHCA/REW, rewarming after deep hypothermic circulatory arrest.

During normothermia epinephrine was associated with a three-fold increase in cTnI phosphorylation, whereas levosimendan maintained cTnI phosphroylation at a physiological ratio (Figure 5). After DHCA, cTnI phosphroylation increased five-fold compared with normothermia. Epinephrine was unable to reverse this increase. On the contrary, this change was reversed by levosimendan, which re-established the physiological cTnI phosphorylation ratio observed during normothermia (Figure 5).

## Discussion

Data presented here clearly show that levosimendan administered after rewarming from DHCA prevents Ca^2+^ desensitization mediated by cTnI phosphorylation. Levosimendan, compared to epinephrine, led to a significantly better preservation of myocardial ATP content as well as energy charge and to a reduction in plasma lactate concentrations. In comparison, Levosimendan was superior in improving myocardial systolic and diastolic functions as demonstrated by PV-analysis.

Clinically deep hypothermia, either accidental or induced as a neuroprotective measure during aortic arch interventions, leads to an overall myocardial depression that aggravates ischemia-reperfusion injury [2]. Although systolic and diastolic dysfunctions are often transient, more permanent injury in the form of necrosis and apoptosis might occur. Pulmonary dysfunction, which is frequently associated with hypothermia-induced myocardial dysfunction, worsens the low cardiac output state [1].

Experimental models of deep hypothermia and rewarming confirm and explain these clinical findings [15,16]. In an experimental animal model of DHCA, left ventricle contractility, relaxation and afterload were markedly, but transiently, depressed early after reperfusion and mildly depressed late after reperfusion [17]. During cooling, end systolic volume decreases, but during the rewarming phase, isovolumetric pressure is depressed and ventricular wall shortening reduced, whereas diastolic function is reported to be affected to a lesser extent. Hence, the observed hypothermia-induced cardiac dysfunction is mainly due to compromised systolic function. Based on recent reports, it is possible to advocate that one of the main underlying mechanisms of hypothermia- rewarming-induced contractile dysfunction could be Ca^2+^ overload, which is defined as mechanical or electrical contractile failure induced by elevated intracellular Ca^2+^ content in myocytes [3,16,18].

Ca^2+^ overload may induce cardiac contractile dysfunction through different pathways. In particular after hypothermia and rewarming, increase in reactive oxygen species, decreased ATP synthesis and damaged mitochondrial ultrastructure have been reported [3].

YS Han and colleagues have observed that papillary muscle twitch force was reduced by 35% after rewarming despite a 15% increase in the intracellular Ca^2+^ content, indicating that reduced contractile force after rewarming is related to a decrease in myofibrillar Ca^2+^ sensitivity rather than impairment in intracellular Ca^2+^ regulation [4]. Significant increases in the temporal parameters of contraction and relaxation in twitch force after rewarming likely reflect a change in myosin/actin cross-bridge cycling kinetics. By plotting phase-plane loops (the relationship between twitch force and intracellular Ca^2+^ concentration), which represent coarse dynamic indices of myofilaments Ca^2+^ sensitivity during the relaxation phase of the twitch force, a rightward shift was observed in the relaxation phase during stable hypothermia, as well as after rewarming, in intact papillary muscle [4]. This indicates a decrease in myofilaments Ca^2+^ sensitivity due to hypothermia and rewarming.

These data propose that hypothermia decreases cardiac myofilaments Ca^2+^ sensitivity, which is not reversed by rewarming, thus contributing to post-hypothermic cardiac contractile dysfunction.

Numerous studies have demonstrated that increased phospho-mediated phosphorylation of the sarcomeric protein cTnI reduces myofilament Ca^2+^ sensitivity and shifts the force-Ca2+ relationship rightward under normothermic circumstances [5,19,20].

One way to increase the contractile force of the sarcomere is to increase cytosolic Ca^2+^; another is to modulate the response of sarcomeres to Ca^2+^. The unique position of cTnI to regulate and modulate cardiac function under the influence of adrenergic signaling is well documented [21]. In more detail, cTnI has a NH2-terminal extension of approximately 30 amino acids that houses Ser23/24, which if phosphorylated by phosphokinase A depresses sarcomere sensitivity to Ca^2+^and increases cross-bridge kinetics.

In the present study, we found that after rewarming from DHCA, there was a significant increase in cTnI phosphorylation at phosphokinase A sites, as shown by Western blot analysis. Phosphorylation of cTnI is mediated by several kinases (phosphokinase A, C, G), but particularly phosphokinase A-mediated phosphorylation via-adrenergic stimulation plays a major physiological role in meeting the circulatory demand in the physiological state [21].

In general, changes in the cTnI phosphorylation status, as reported in the failing human heart are considered a change in the balance between kinase and phosphatase activities, and the optimal balance is realized during normal cardiac function. Thus, hypothermia and rewarming, depending on severity and depth, appear to tip the balance between kinase and phosphatase activities, leading to alterations in myofilament Ca^2+^ sensitivity and myocardial contractility.

Furthermore, we have previously reported a lack of effect of pharmacological agents to support cardiac contractile function mediated via the receptor - cAMP - phosphokinase A pathway during, as well as after, rewarming [11]. Likewise, if hypothermia and rewarming *per se* activate the receptor - cAMP - phosphokinase A pathway, one may conclude that an additional pharmacological stimulus of the receptor under these circumstances would achieve a markedly reduced cardiac inotropic effect.

Moreover, cardiovascular effects mediated by β-adrenoceptors are significantly diminished after hypothermia, suggesting that the use of the β-adrenoceptor specific agonist should be reconsidered [7-9]. Additionally, pharmacologic therapy with catecholamines has substantial limitations because these are associated with Ca^2+^ overload, elevated myocardial oxygen consumption, arrhythmogenesis and regional hypoperfusion leading to organ damage [8-10].

Levosimendan is a pyridazinone dinitrite derivative belonging to the new inotropic drugs class of Ca^2+^ sensitizers. It exerts its positive inotropic effect by sensitizing cardiac troponin C to calcium during systole, thus increasing cardiac performance without increasing myocardial oxygen consumption. Levosimendan also has a weak arrhythmogenic effect [22]. Our group demonstrated for the first time the efficacy of levosimendan in improving myocardial dysfunction after deep hypothermia-rewarming [11].

This study was conducted in a previously described and validated rat model of CPB [12]. DHCA and CPB-assisted rewarming reproduces the clinical setting of both cardiac surgery interventions and extracorporeal resuscitation after accidental hypothermia. The results of this study demonstrated that levosimendan treatment after rewarming produced a significant improvement of clinically used parameters of cardiac functions (HR, MAP, SV and CO). In the same manner, more precise parameters derived from pressure-volume analysis being insensitive to preload effects confirmed significantly improved systolic function after levosimendan treatment when compared with epinephrine.

Epinephrine produced an increased MAP without an improvement in SV. This increase in afterload during the rewarming phase may explain the significantly higher lactate plasma levels with epinephrine compared to levosimendan after rewarming from hypothermia. Moreover, levosimendan was superior in improving diastolic function, which is clearly evident from the left ventricular relaxation indexes. In line with these findings, levosimendan also significantly better preserved high-energy phosphates shown by energy charge and ATP myocardial content than epinephrine. The differences concerning phosphocreatine, hypoxanthine and xanthine between groups are due to the high standard deviation values (control- and epinephrine group; Table 2) not significant. However, the mean values are lowest for phosphocreatine and highest for xanthine in the epinephrine group. In addition, there was a significant rise of AMP contents of the hearts in the epinephrine group compared to the control - as well as levosimendan group. These findings are in line with the observed significantly increased arterial lactate levels after treatment with epinephrine.

Energy charge uses the total measured concentrations of AMP, ADP and ATP determined after careful digestion (liquid nitrogen, ball mill and neutralization of then sample) by HPLC and, therefore, do not directly reflect the bioenergetic situation of the free metabolites in the cytosol. The assessment of free fluctuating concentrations of these metabolites would require 31P NMR and a number of near equilibrium expressions using the creating kinase and adenylatekinase equilibrium [23]. However, energy charge as calculated value from the absolute concentrations of AMP, ADP and ATP has been proven to be a valuable parameter for energetic situations of cells and tissue [24-26].

In the present study and differently from the previously reported [11], there were no major arrhythmic events in the epinephrine-treated group or in levosimendan-treated one. No other downsides related to treatment were documented.

Further investigations are needed to validate these results in the clinical setting and to evaluate the cost/benefit ratio in this setting of high-risk patients. Nonetheless, on the basis of these findings, use of Ca^2+^ sensitizer drugs, such as levosimendan, seems rational in induced or accidental deep hypothermia.

Finally, some important limitations remain. The animal model does not faithfully reproduce the clinical setting. Therefore, the outcome of this study in a rat model of CPB remains to be demonstrated in large-animal and clinical studies. However, because of the small animal size, this model allows rapid and precise control over body temperature, which is difficult to achieve in clinical practice at present, and it may be particularly appropriate for defining optimal hypothermia parameters. Another limitation of the present study is that we only investigated a single dose of epinephrine and levosimendan. One, therefore, cannot rule out the possibility that different dosages or treatment schemes might have generated different functional outcomes from the one herein. Nevertheless, the particular infusion rate for levosimendan and for epinephrine was deliberately chosen because it is the highest dose regimen suggested in the clinical setting.

## Conclusion

Levosimendan due to prevention of calcium desensitization by cTnI phosphorylation is more effective than epinephrine for treatment of myocardial dysfunction after rewarming from DHCA.

## Key messages

•CPB-assisted rewarming after DHCA results in acute myocardial dysfunction.

•Levosimendan but not epinephrine prevents myofilaments Ca^2+^ desensitization mediated by cTnI phosphorylation after rewarming.

•In this setting, levosimendan is more effective than epinephrine in improving systolic and diastolic functions, high-energy phosphates myocardial content and in reducing lactate plasma concentrations.

## Abbreviations

CO: Cardiac output; CPB: Cardiopulmonary by-pass; cTnI: Cardiac troponin I; DHCA: Deep hypothermic circulatory arrest; EDPVR: End-diastolic pressure-volume relationship; Ees: Slope of left ventricular end-systolic pressure volume relationship; HPLC: High-performance liquid chromatography; HR: Heart rate; MAP: Mean arterial pressure; PRSW: Preload recruitable stroke work; SV: Stroke volume; +dP/dt: Maximal slope of the systolic pressure increment; -dP/dt: Maximal slope of diastolic pressure decrement.

## Competing interests

The authors declare that they have no competing interests.

## Authors’ contributions

AR, AM and GF designed the study. AR, DL, EM and MT performed the experiments. SH, AG and TS performed biochemical analysis. AR, SH, GBL and TS analyzed the results. AR, SH, GBL, TS, AM and GF wrote and corrected the manuscript. All authors read and approved the final manuscript.
